# Radiographic features of Ollier’s disease – two case reports

**DOI:** 10.1186/s12880-017-0230-8

**Published:** 2017-12-02

**Authors:** Jamshid Sadiqi, Najibullah Rasouly, Hidayatullah Hamidi, Salahuddin Siraj

**Affiliations:** 1 Radiology Department of French Medical Institute for Mothers and Children (FMIC), Kabul, Afghanistan; 2 Orthopedic Department of French Medical Institute for Mothers and Children (FMIC), Kabul, Afghanistan; 3 Radiology Department, French Medical Institute for Mothers & Children (FMIC), behind Kabul Medical University Aliabad, P.O. Box: 472, Kabul, Afghanistan

**Keywords:** Multiple enchondromatosis, Enchondromas, Tubular bones, Metaphysis, Bone deformity, Conventional radiography

## Abstract

**Background:**

Ollier’s disease is a non-hereditary, benign bone tumor which is usually characterized by presence of multiple radiolucent lesions (enchondromas) in the metaphysis of long bones with unilateral predominance. The disease is a rare clinical entity with 1/100000 occurrence in early childhood. Patients mostly present with multiple hard swellings and deformity of the tubular bones specially hands and feet with leg discrepancy and pathologic fractures.

**Case presentation:**

We present two cases of Ollier’s disease in a 13 years old female and 8 years old boy which had no specific symptoms. The girl had multiple hard swellings and deformity in the fingers of both hands and left toes with left leg deformity and discrepancy. Her plain radiographs demonstrated multiple expansile enchondromas in the phalanges of hands, left toes and metaphyses of upper humeri as well as left leg bones. The enchondromas were also noted in the left iliac bone and anterior end of ribs. The boy had bowing deformity and shortage of left leg with multiple enchondromas in the metaphyses of left femur, left tibia and fibula as well as left iliac bone in his radiographic images.

**Conclusion:**

Ollier’s disease is usually diagnosed by clinical signs and typical location of enchondromas across skeleton in conventional radiography. It usually does not need specific treatment. Well understanding of the clinical manifestation and radiographic features can prevent unnecessary application of other imaging modalities; while other diagnostic imaging modalities like MRI, ultrasound and scintigraphy can be used in complicated and painful conditions.

## Background

Multiple enchondromatosis is a rare bone disease with six subtypes. Ollier’s disease is a non-hereditary subtype of the disease which for the first time was introduced by Ollier in 1899 [1, 2]. It is characterized by presence of at least three enchondromas in the appendicular bones. The disease prevalence is 1/100000 of population with almost equal sex distribution and early manifestation in first decade of life [1–3]. The patients usually present with multiple hard swellings in the fingers and toes associated with asymmetric deformity of the extremities [4]. The plain radiographs of the affected bones demonstrate various number, size and location of enchondromas in the metaphyses of tubular bones with extension into the diaphyseal and epiphyseal regions [2]. Some of these enchondromas may show expansile behavior called enchondromas protuberance. Clusters of enchondromas cause widening of the metaphyseal regions. In the lower extremities the lesions may result in bone deformity and limb discrepancy. Punctate calcifications with light trabeculations are also seen in the metaphysis of long bones [5]. Sometimes Pelvis bone especially the iliac crest is involved but sternum, ribs and skull bones are rarely affected [1]. In one of the present cases, the enchondromas exist in the anterior ribs representing a rare location of the disease.

## Case presentation

### First case

**Fig. 1:**
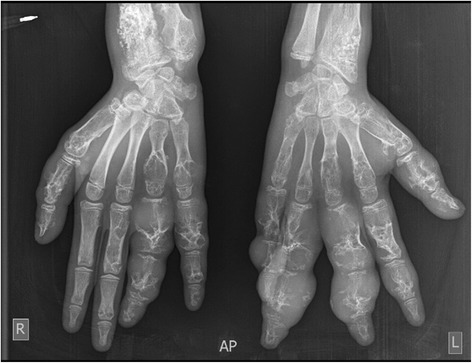
Multiple expansile lytic lesions in the metacarpi and phalanges of both hands with sparing of right index finger and lytic lesions in the right distal radius and ulna with sparing of the left ulna

**Fig. 2:**
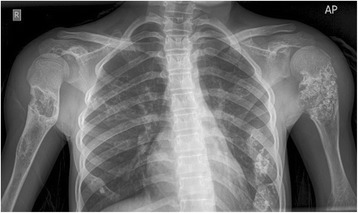
AP chest x-ray shows multiple lytic lesions with punctate calcifications in the anterior end of ribs and proximal metaphyses of both humeri extending to the diaphysis of right humerus

**Fig. 3:**
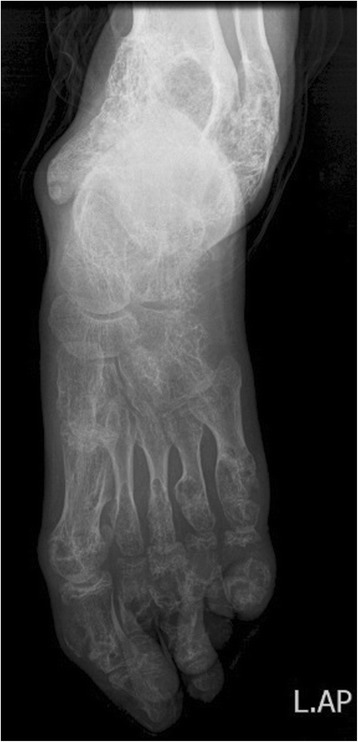
Anterior posterior x-ray of left foot: Multiple expansile lytic lesions in the metatarsi and toes of left foot with bone deformity and lytic lesions in the metaphysis of distal tibia and fibula

**Fig. 4:**
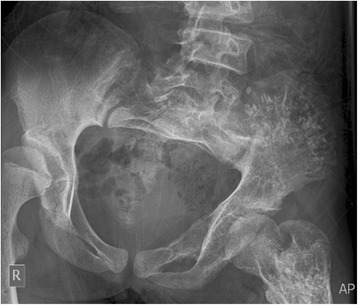
Multiple lytic lesions with punctate calcifications in the left iliac bone and proximal metaphysis of left femur

**Fig. 5:**
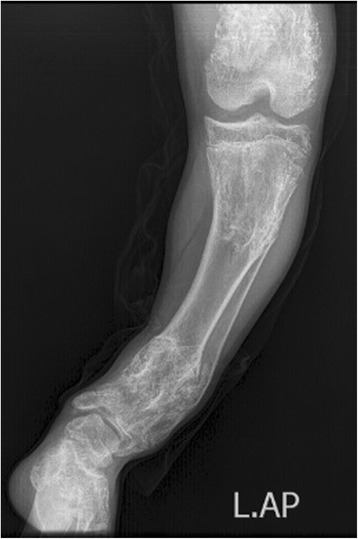
Anterior posterior x-ray of left leg: lytic lesions in the proximal and distal metaphyses of left tibia and fibula

A-13 year old girl was referred to the radiology department of French Medical Institute for Mothers and Children (FMIC) for taking plain radiographs of the axial and appendicular bones. The patient had short left leg with abnormal swelling and deformity of fingers and left toes. The swelling were hard in palpation however no pain was noticed. The overlying skin appeared normal. In radiographic images, multiple expansile lytic lesions (enchondromas) were noted in the metacarpi and phalanges of hands with sparing of the index finger of right hand associated with multiple lytic lesions in the distal ulna and radius of both hands with sparing of left distal ulna (Fig. 1). Chest plain image demonstrated multiple enchondromas in the anterior end of the ribs bilaterally as well as proximal metaphyses of both humeri, extending into right humerus diaphysis with multiple punctate like calcifications (Fig. 2). Enchondromas were also noted in the metatarsi and toes of left foot, left distal tibia and fibula in plain image of left foot (Fig. 3). Multiple lytic lesions with punctate calcifications were also seen in the left iliac bone and proximal metaphysis of left femur in anterior posterior radiograph of pelvis bone (Fig. 4). Plain image of left calf showed multiple foci of calcifications with lytic lesion in the left distal femur as well as proximal and distal metaphyses of left tibia and fibula (Fig. 5).

### Second case

**Fig. 6:**
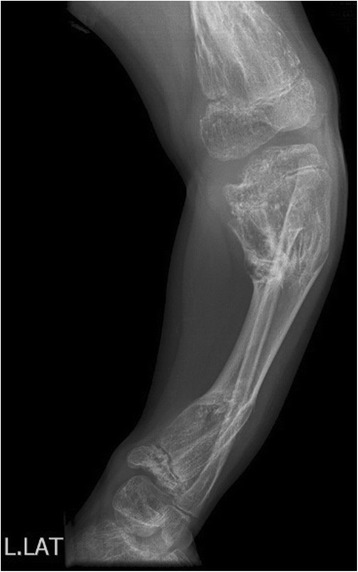
Lateral radiograph of left lower limb: bone deformity with multiple lytic lesions in the upper and lower metaphyses of tibia and fibula as well as distal end of femur

**Fig. 7:**
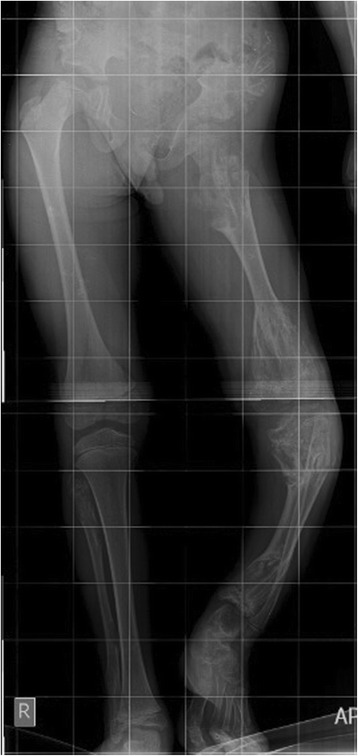
Anterior-posterior radiograph of pelvis and both lower limbs: enchondromas in the upper and lower ends of left femur, tibia, fibula as well as left iliac crest with left lower limb discrepancy and bowing deformity

An 8 year- old boy was referred to our radiology department for taking radiographs of left leg and pelvis bones. The patient had left leg discrepancy with bowing deformity without specific pain. The lateral radiograph of the left leg demonstrates bowing deformity with enchondromas in the distal femur as well as proximal and distal ends of left tibia and fibula (Fig. 6). The anterior posterior radiograph of pelvis and both lower extremities showed lytic lesions in the proximal and distal ends of left femur, tibia, fibula and left iliac bone with deformity and significant left leg discrepancy (Fig. 7).

## Discussion and conclusion

The etiology of Ollier’s disease is not well known however abnormal signaling pathways in the proliferation and differentiation of chondrocytes have been proposed which results in the development of intraosseous cartilaginous foci. Some authors have indicated genetic aberrations like heterozygous mutations of PTHR1, IDH1 and IDH2 genes as the pathogenesis of the disease [3]. The disease has specific radiographic features representing unilateral involvement with presence of multiple lytic lesions in the center of tubular bones of hand, foot and long bones particularly in metaphyseal regions. Sometimes there is symmetrical involvement but still with unilateral predominance. Occasionally during the course of the disease pathologic fractures may occur due to the cortical thinning [2]. Ultrasound, Magnetic Resonance Imaging (MRI) and Scintigraphy are used when the lesions increase in size and causing pain. MRI shows lobulated lesions in the affected bones returning intermediate signal intensity in T1 with intermediate to high signal intensity in T2 weighted images [1]. In bone scan multiple foci of radiotracer uptakes are appreciated mainly in the tubular bones [2].The complication of enchondromatosis is its malignant transformation into the chondrosarcoma which can result in cortical erosion and infiltration into the adjacent soft tissues with indistinguishable and irregular outline of the tumor surface [4]. The differential diagnosis of the Ollier’s disease is hereditary multiple exostosis in which the lesions are usually located in the surface of bones rather than in the center (lesions are located in the center of the bones in enchondromatosis) [1]. Another type of multiple enchondromatosis is called Mafucci syndrome which is characterized with presence of multiple enchondromas associated with soft tissue hemangiomas and occasionally lymphangiomas [4]. As the diagnosis of multiple enchondromatosis is based on the clinical features and specific radiographic manifestations therefore the histopathologic examinations are not usually preformed. In cases when malignant transformation is suspected histopathologic exams are necessary. The literature has indicated a 35% progression into the chondrosarcomas [6, 7]. Asymptomatic patients of multiple enchondromatosis do not need medical or surgical treatments but in cases of growth defects, pathologic fractures and malignant transformation surgical intervention is performed [4]. Radiotherapy and chemotherapy are also advised in cases of malignant transformation [8]. It is concluded that Ollier’s disease is a benign bone tumor which is well diagnosed by its unique clinical and radiographic manifestations.
